# Supplementary material for: The pluripotency factor NANOG contributes to mesenchymal plasticity and is predictive for outcome in esophageal adenocarcinoma
Source: Commun Med (Lond). 2024 May 17;4:89. doi: 10.1038/s43856-024-00512-z (PMC11101480; doi:10.1038/s43856-024-00512-z)
Supplement: Supplementary file 1 — Supplemental material [file 43856_2024_512_MOESM1_ESM.pdf]

SUPPLEMENTARY INFORMATION

SUPPLEMENTARY FIGURES

Supplemental Figure S1. Cell line responses to chemoradiation

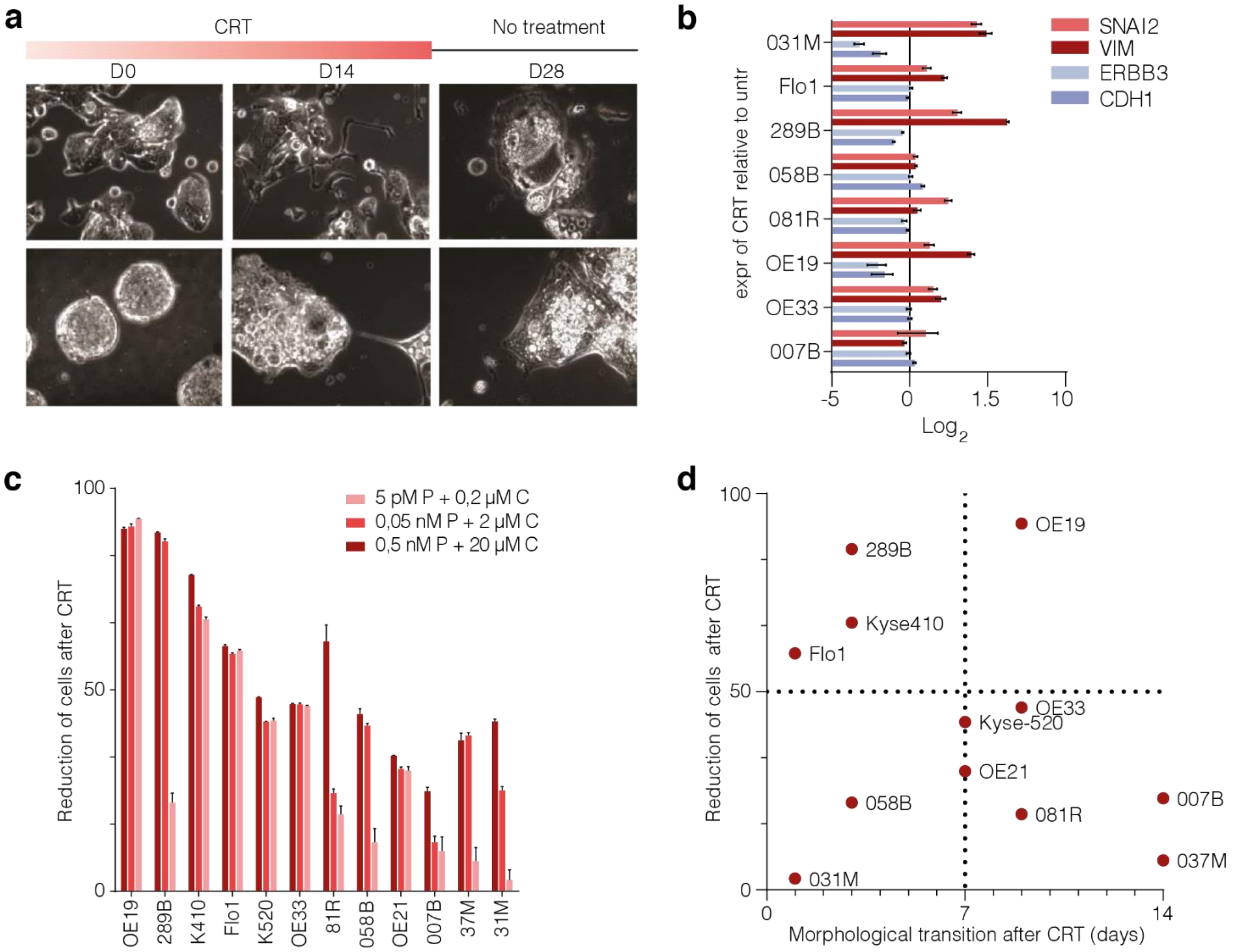

(a) After the CRT regimen for 14 days, cells were left untreated until day 28 and assessed by phase-contrast imaging.

(b) All EAC lines were treated with CRT for 14 days and gene expression was determined using quantitative RT-qPCR. Same ranking of cell lines as seen in Figure 1f, EAC only. Bar graphs show means of technical triplicates ±SD. Statistical test is unpaired Student's t-test, * $p < 0.05$, ** $p < 0.01$, *** $p < 0.001$, **** $p < 0.0001$.

(c) Panel of esophageal cell lines treated with chemotherapy for 14 days. Reduction of cells measured as absolute Crystal Violet absorbance at 600nm. Panel plated and measured at the same time point, means of technical triplicates ±SD. P = paclitaxel, C = Carboplatin.

(d) Reduction of cells after chemotherapy as measured in B (concentration 0,05 nM P + 2µM), means of technical triplicates ±SD, plotted against morphology-based ranking as established in Figure 1c.

Supplemental Figure S2. Ridge regression models

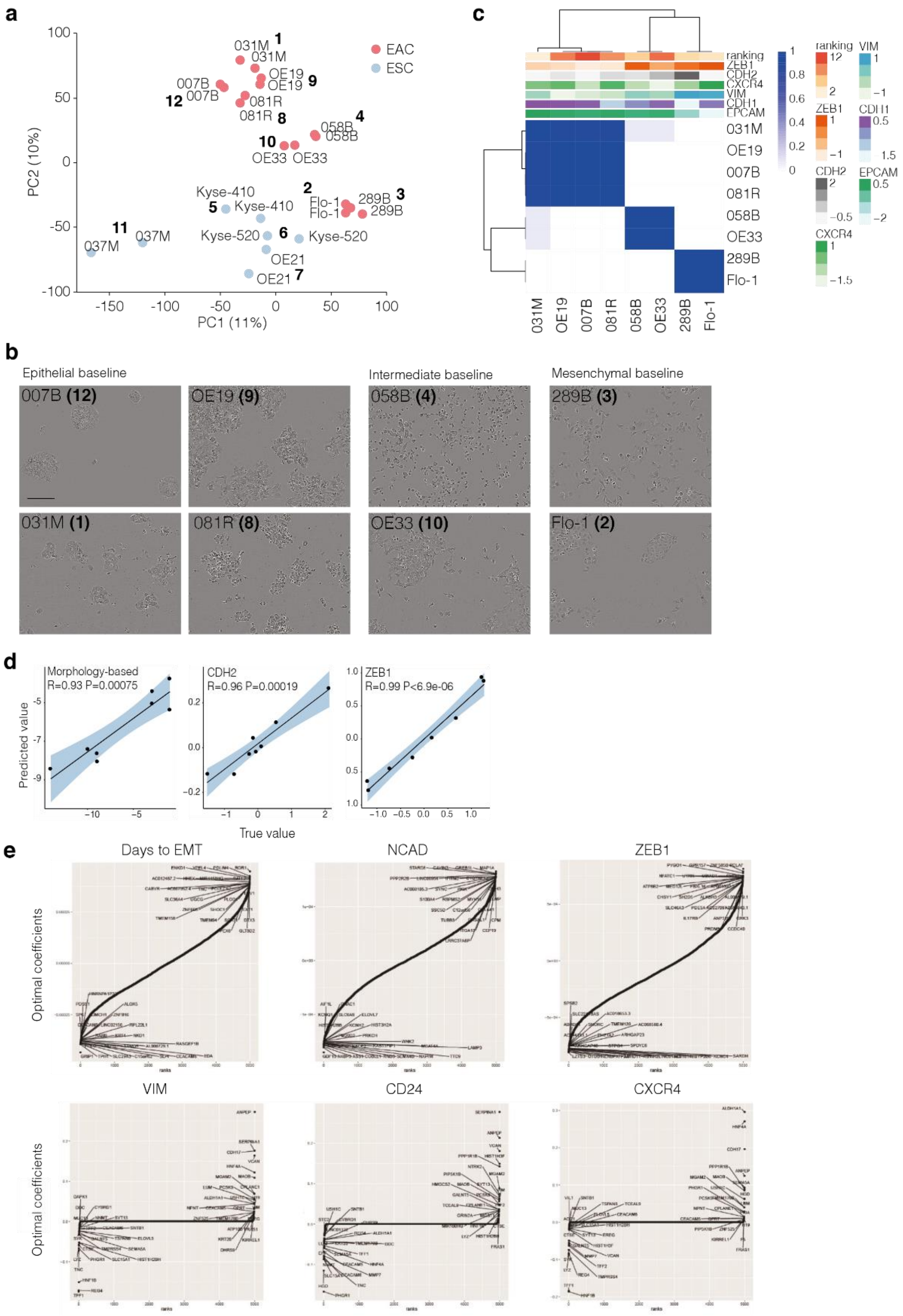

(a) Principal component analysis (PCA) plot showing cell line panel samples in biological replicates. Dim = Dimensionality.

(b) Phase-contrast images of baseline phenotype of the panel of eight EAC cell lines including the visually scored epithelial to mesenchymal state.
(c) Consensus matrix at k=3 depicts three distinct cluster groups: epithelial, intermediate, and mesenchymal. Consensus values within this matrix span a spectrum from 0 (indicating that elements are never clustered together) to 1 (signifying that they are always clustered together), represented by a gradient from white to dark blue. These results consistently align with the grouping observed in panel (b). Plotted above are canonical epithelial and mesenchymal markers. Plasticity ranking is shown in top row. Note that darker color indicates slower EMT onset.
(d) Ridge regression prediction correlations of predicted and true value in the three indicated *in vitro* markers with RNAseq expression data by leave-one-out-cross validation (LOOCV).
(e) Ridge regression model coefficients per model. Indicated names are top 25 positive and negatively correlating genes for each *in vitro* model.

Supplemental Figure S3. Selection of pre-treatment biopsies for RNA-sequencing

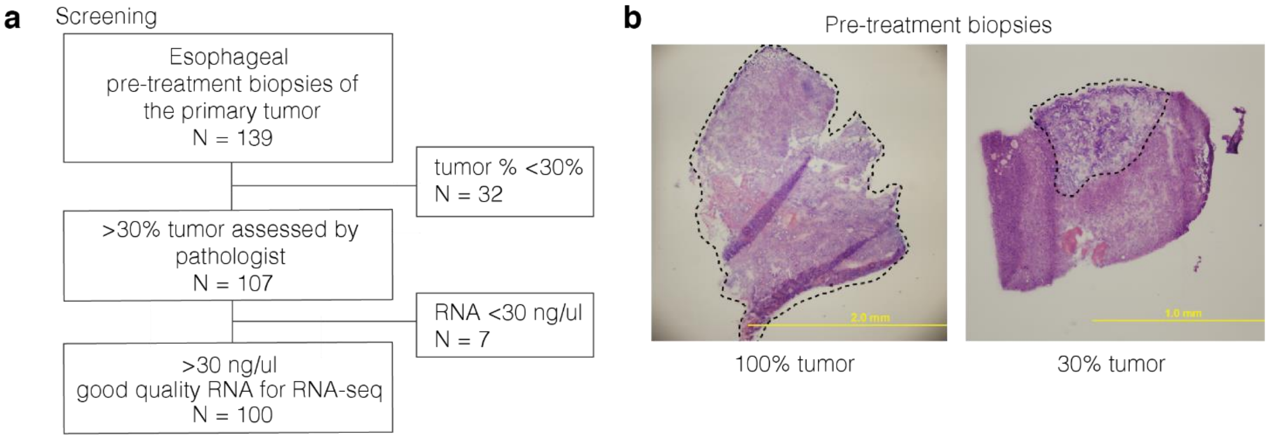

(a) Flow diagram of processed biopsies for RNA-sequencing. Patient esophageal pre-treatment biopsy screening and selection. Samples were excluded due to reasons indicated.
(b) Example of Hematoxylin and Eosin (H&E) staining of two patient biopsies (AMC-007-EAC and AMC-057-EAC) for assessment of tumor percentage of the total tissue slide by a trained pathologist. Black dotted line indicates tumor cell area.

Supplemental Figure S4. NFE2L2 not predictive for outcome in pre-treatment biopsies

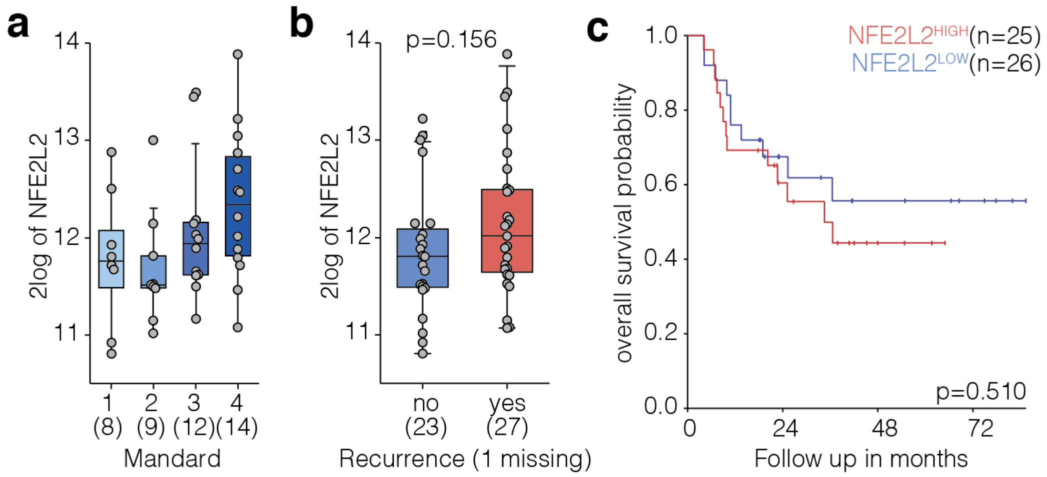

(a) Box-dot plot of *NFE2L2* expression for pre-treatment EAC biopsies concurrent with available chemoradiation response assessed in resection specimen after CRT by Mandard score. Samples sizes indicated and individual data points are shown. Mandard 1 (no tumor left after CROSS), Mandard 2 (major response), Mandard 3 (medium response) and Mandard 4 (minor response). Box indicates mean and interquartile range (IQR), whiskers 1.5x IQR. Patients per group indicated.
(b) Box-dot plot of *NFE2L2* expression in pre-treatment EAC biopsies of patients with or without recurrence after CRT treatment and resections. Samples sizes indicated. Box indicates mean and IQR, whiskers 1.5x IQR. Mann-Whitney U statistical test. Patients per group indicated.
(c) Kaplan-Meier survival analysis in all pre-treatment EAC biopsies with known patient follow-up data. Biopsies were dichotomized by median expression of *NFE2L2*. Survival analysis was performed using Kaplan-Meier analysis and Log-rank statistical test. Patients per group indicated.

Supplemental Figure S5. Pharmacological inhibition of pluripotency combined with chemoradiation

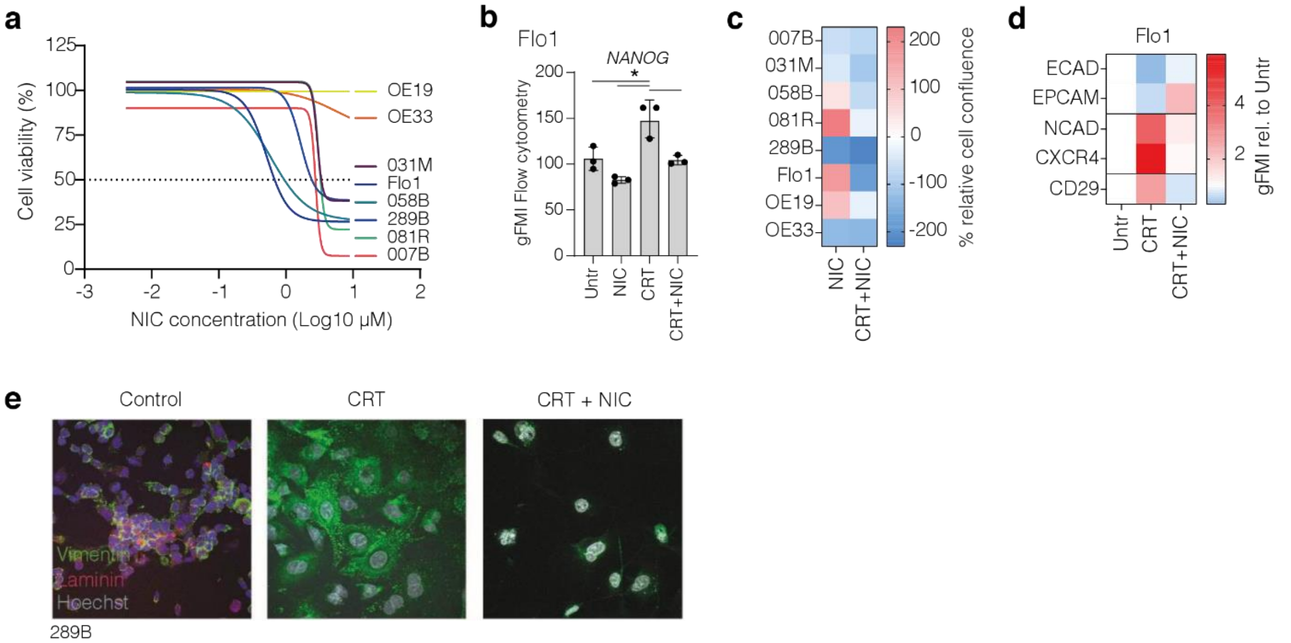

(a) Indicated EAC lines were treated with a concentration dosis of Niclosamide. Viability of cells assessed after 72h treatment by Cell Titer Blue assay. Panel plated and measured at the same time point, average of technical triplicates per concentration, fit-curved.

(b) Flow cytometry gMFI of NANOG protein in Flo1 cells. Technical triplicates ±SD, Mann–Whitney U test between , * $p < 0.05$.

(c) Relative confluence (%) of EAC cells treated with CRT for 14 days, with or without Niclosamide, concentration determined per cell line as the IC_{50}. Average confluence of technical triplicates.

(d) Relative gMFI of epithelial (EpCAM, ECAD) and mesenchymal (CXCR4, NCAD, CD29) markers in Flo1 cells treated with CRT, Niclosamide or combined, compared to untreated EAC cells. Average gMFI of technical triplicates.

(e) 289B cells as in panel c were processed for immunofluorescence for vimentin (green), Laminin (red), and nuclei (Hoechst; blue). Magnifications and laser settings were identical between all images. Scale bars are 50 µm.

Supplemental Figure S6. Pluripotency inhibition combined with radiation in preclinical EAC models

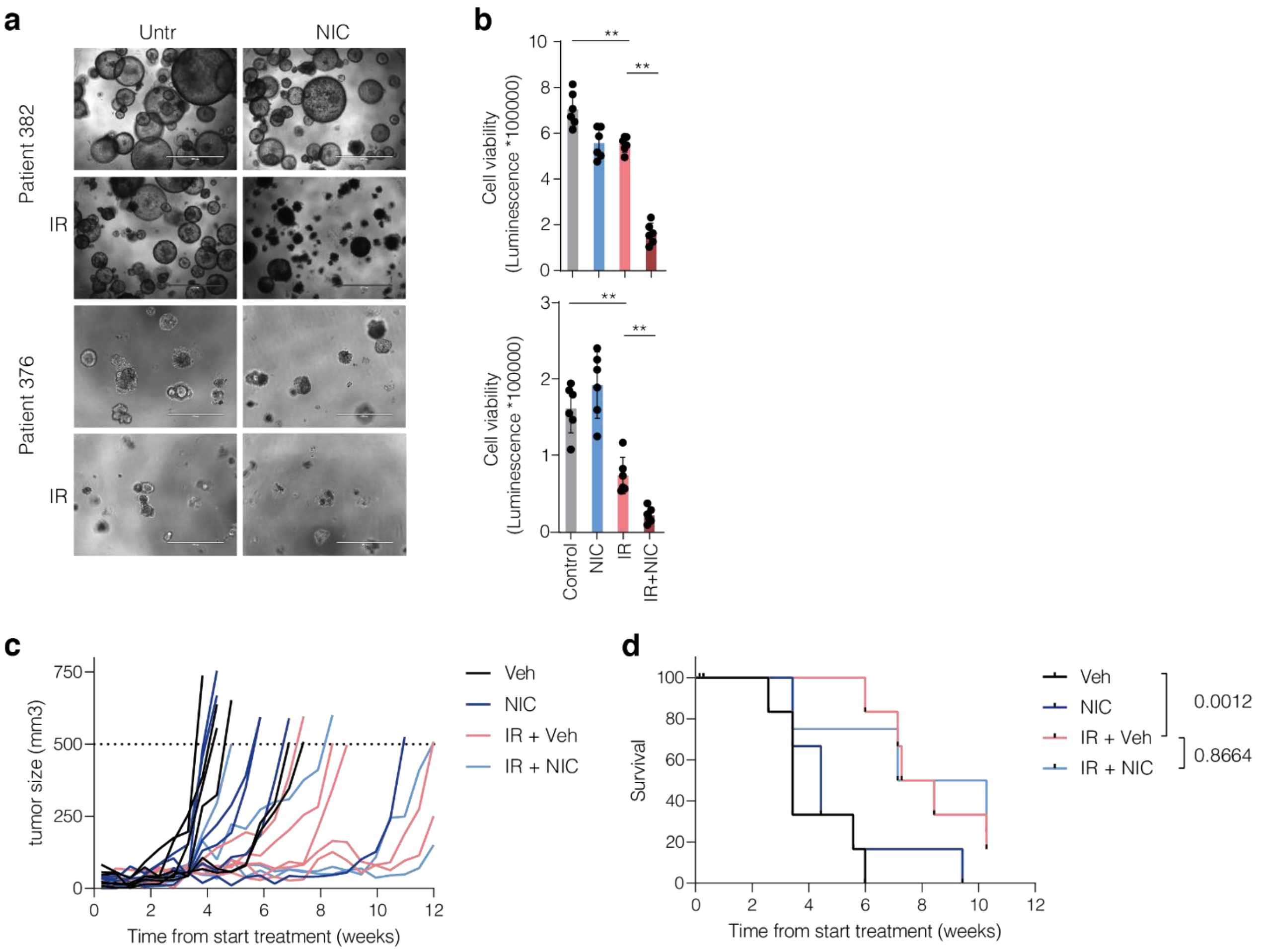

(a) Patient-derived EAC organoid cultures 382 and 376 were exposed to radiation with 0.33 uM Niclosamide for 5 days. Outgrowth was monitored by phase-contrast microscopy.

(b) Organoids as in panel a were treated for 5 days. Viability was assessed with Cell Titer Glow in 96-well plates. Luminescence was measured after 3 hours. Graphs show 6 biological replicates with mean ±SD. Statistical test performed is unpaired Student's t-test, ** $p < 0.01$.

(c) Tumor volumes over time from start of injection for each treatment group. Lines indicate individual mice. Group size N=6, mice that reached a humane endpoint other than tumor volume 500mm^3 were excluded, resulting in N4-5 per group for analysis. On days of radiation mice also received oral gavage with vehicle (1% DMSO, 1% Tween80 in 98% PBS) or Niclosamide (50mg/kg in vehicle).

(d) Kaplan-Meier survival analysis of mice. Events are humane endpoints by maximum tumor growth. Group size N=6, censured mice indicated in black short lines. p-values were computed by log rank test and indicated for groups of interest.

Supplemental Figure S7. *shNANOG* and *shOCT4* cells show reduced mesenchymal plasticity

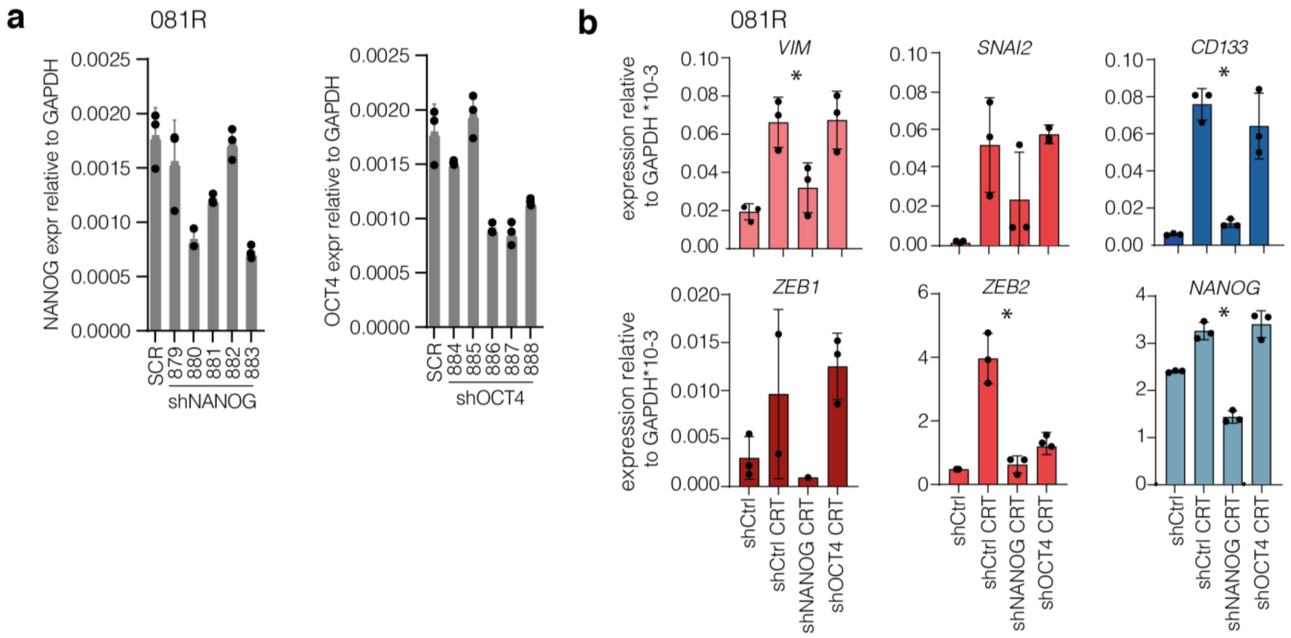

(a) Validation of *NANOG* and *OCT4* knockdown constructs in 081R cells with quantitative RT-PCR. Technical triplicates are represented as mean ±SD relative to RPS18.

(b) 081R cells were treated with CR for 14 days and gene expression was determined with quantitative RT-PCR. Bar graphs show means of technical triplicates ±SD. Statistical test is unpaired Student's t-test, * $p < 0.05$, ** $p < 0.01$, *** $p < 0.001$, **** $p < 0.0001$.

Supplemental Figure S8. Silencing *NRF2* does not prevent EMT but does sensitize to chemoradiation

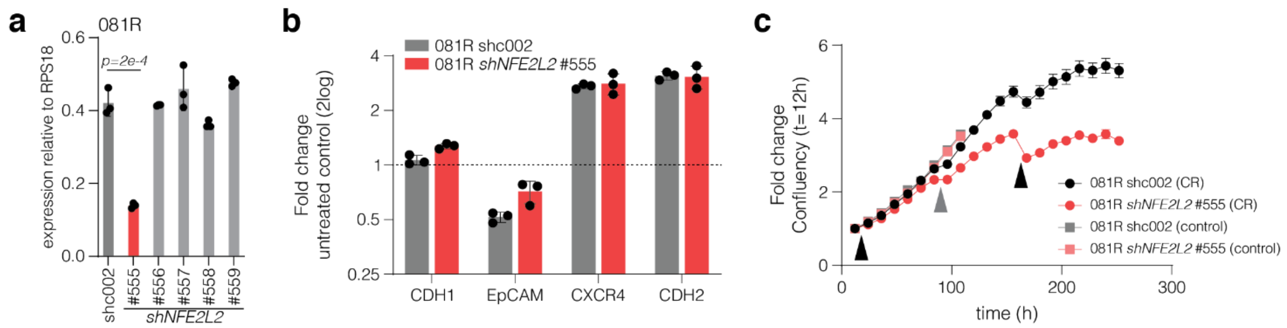

(a) *NFE2L2* (NRF2) gene expression was measured by RT-qPCR after transducing with *NFE2L2* -targeting shRNA or scrambled control (shc002) and puromycin selection. Bar graphs show means of technical triplicates ± SD. Statistical test is unpaired Student's t-test.

(b) Fold change of epithelial and mesenchymal markers after 2 weeks of chemoradiation with *NFE2L2*-silenced 081R cells and scrambled controls. Bar graphs show means of biological triplicates ± SD.

(c) Confluence over time relative to t=12h after seeding. Cells in the chemoradiation (CR) group were treated from two weeks: the black arrows indicate the start of the treatment with carboplatin, paclitaxel and daily radiation, and the gray arrow indicate the start of the drug holiday. Dots show means of biological triplicates ± SD.

SUPPLEMENTARY TABLES

Supplemental Table S2. Cell line panel baseline characteristics.

ID	Plasticity ranking	Donor ID	Sex	Tissue source	Exposed to treatment	Age	cTNM	Grade	Tumor location
007B	12	AMC-007-EAC	F	Biopsy	No	50	cT3N1M0	2	Gastroesophageal junction/cardia
031M	1	AMC-031-EAC	M	Metastasis	No	78	cT3N3M1	3	Distal esophagus
037M	11	AMC-037-ESC	M	Metastasis	No	68	cTxN2M1	*NA*	Mid esophagus
058B	4	AMC-058-EAC	M	Biopsy	No	76	cT3N2M0	*NA*	Gastroesophageal junction/cardia
081R*	8	AMC-081-EAC	M	Resection	dCRT	38	cT3N1M1	3	Distal esophagus
289B	3	AMC-289-EAC	M	Biopsy	No	51	cT3N0M0	3	Gastroesophageal junction/cardia
Flo-1	2	–	M	*NA*	*NA*	68	–	–	–
OE19	9	–	M	Resection	No**	72	–	–	–
OE33	10	–	F	Resection	No**	73	–	–	–

* Patient AMC-081-EAC received definitive chemoradiation (dCRT) and underwent a salvage resection after. From this resection tissue 081R cell line was derived.

** OE19 and OE33 were successfully established in 1996 and therefore unlikely given therapy before resection and cell line isolation.

Note that only for 007B a Mandard score is available (Mandard 4). For the other cell lines the source is biopsy or metastasis, for the 289B Mandard score is missing.

Supplemental Table S3. Primer sequences used for qRT-PCR.

Gene	Forward	Reverse
GAPDH	5’-AATCCCATCACCATCTTCCA -3’	5’- TGGACTCCACGACGTACTCA-3’
VIM	5’-CCCTCACCTGTGAAGTGGAT-3’	5’-TCCAGCAGCTTCCTGTAGGT-3’
SNAI2	5’-GGTCAAGAAGCATTTCAACG-3’	5’-CACAGTGATGGGGCTGTATG-3’
CDH1	5’-TCTCTGCTCGTGTTTGACTATG-3’	5’-GTCATAGTCCTGGTCTTTGTCTG-3’
CD44	5’-TGGAGCAAACACAACCTCTG-3’	5’-CCACTTGGCTTTCTGTCCTC-3’
ERBB3	5’-TGGGGAACCTTGAGATTGTG-3’	5’-GAGGTTGGGCAATGGTAGAG-3’
CD133	5’-TCCACAGAAATTTACCTACATTGG-3’	5’-CAGCAGAGAGCAGATGACCA-3’
LGR5	5’-ACCAGACTATGCCTTTGGAAAC-3’	5’-TTCCCAGGGAGTGGATTCTAT-3’
TUBB3	5’-CCTGACAATTTCATCTTTGGTCAGAGT-3’	5’-GCACCACATCCAGGACCGAAT-3’
TWIST	5’-GGCATCACTATGGACTTTCTCTATT-3’	5’-GGCCAGTTTGATCCCAGTATT-3’
ZEB1	5’-GCACAAGAAGAGCCACAAGTA-3’	5’-GCAAGACAAGTTCAAGGGTTC-3’
ZEB2	5’-TTCCTGGGCTACGACCATAC-3’	5’-TGTGCTCCATCAAGCAATTC-3’
CDH2	5’-ACAGTGGCCACCTACAAAGG-3’	5’-CCGAGATGGGGTTGATAATG-3’
RPS18	5’-AGTTCCAGCATATTTTGCGAG-3’	5’-CTCTTGGTGAGGTCAATGTC-3’
NANOG	5’-CCTGTGATTTGTGGGCCT G-3’	5’-GACAGTCTCCGTGTGAGGCAT-3’
POU5F1	5’-GTGGAGGAAGCTGACAACAA-3’	5’-ATTCTCCAGGTTGCCTCTCA-3’
c-MYC	5’-AGCTGCTTAGACGCTGGATTTT-3’	5’-TTCCTGTTGGTGAAGCTAACGTT-3’
