# Supplementary material for: The pluripotency factor NANOG contributes to mesenchymal plasticity and is predictive for outcome in esophageal adenocarcinoma
Source: Commun Med (Lond). 2024 May 17;4:89. doi: 10.1038/s43856-024-00512-z (PMC11101480; doi:10.1038/s43856-024-00512-z)
Supplement: Supplementary file 2 — Description of Additional Supplementary Files [file 43856_2024_512_MOESM2_ESM.pdf]

Description of Additional Supplementary Files

File Name: Supplementary Data 1
Description: Patient characteristics of RNA sequenced biopsies

File Name: Supplementary Data 2
Description: Source data underlying graphs in this manuscript
