# Supplementary material for: The pluripotency factor NANOG contributes to mesenchymal plasticity and is predictive for outcome in esophageal adenocarcinoma
Source: Commun Med (Lond). 2024 May 17;4:89. doi: 10.1038/s43856-024-00512-z (PMC11101480; doi:10.1038/s43856-024-00512-z)
Supplement: Supplementary file 6 — Reporting Summary [file 43856_2024_512_MOESM6_ESM.pdf]

nature portfolio

Corresponding author(s): Amber van der Zalm, Mark Dings, Maarten Bijlsma

Last updated by author(s): Oct 17, 2023

Reporting Summary

Nature Portfolio wishes to improve the reproducibility of the work that we publish. This form provides structure for consistency and transparency in reporting. For further information on Nature Portfolio policies, see our Editorial Policies and the Editorial Policy Checklist.

Statistics

For all statistical analyses, confirm that the following items are present in the figure legend, table legend, main text, or Methods section.

n/a	Confirmed	
☐	☒	The exact sample size (n) for each experimental group/condition, given as a discrete number and unit of measurement
☐	☒	A statement on whether measurements were taken from distinct samples or whether the same sample was measured repeatedly
☐	☒	The statistical test(s) used AND whether they are one- or two-sided<br>*Only common tests should be described solely by name; describe more complex techniques in the Methods section.*
☒	☐	A description of all covariates tested
☐	☒	A description of any assumptions or corrections, such as tests of normality and adjustment for multiple comparisons
☐	☒	A full description of the statistical parameters including central tendency (e.g. means) or other basic estimates (e.g. regression coefficient) AND variation (e.g. standard deviation) or associated estimates of uncertainty (e.g. confidence intervals)
☐	☒	For null hypothesis testing, the test statistic (e.g. F, t, r) with confidence intervals, effect sizes, degrees of freedom and P value noted<br>*Give P values as exact values whenever suitable.*
☒	☐	For Bayesian analysis, information on the choice of priors and Markov chain Monte Carlo settings
☒	☐	For hierarchical and complex designs, identification of the appropriate level for tests and full reporting of outcomes
☐	☒	Estimates of effect sizes (e.g. Cohen's d, Pearson's r), indicating how they were calculated

Our web collection on statistics for biologists contains articles on many of the points above.

Software and code

Policy information about availability of computer code

Data collection	Cell lines and primary cultures in duplicate (N=24) and 78 EAC biopsies were processed for RNA-sequencing. Library preparation was performed using Total RNA library prep RiboErase (Roche, Basel, Switzerland). Samples were sequenced in three batches on an Illumina HiSeq4000 with single 50 bp reads and 100 million reads per sample. All sequencing data were quality-controlled using FastQC42 and found to be of high quality. RNA-Seq reads were aligned to the human reference genome (NCBI37/hg19) using using STAR v2.7.1 and annotated with Gencode v32, retaining only uniquely mapped reads. The resulting gene expression profiles were converted into DESeq2_vst values using DESeq2 and log2-transformed. Non-biological batch effects were examined using PCA, and RUVg corrections were applied. Subsequent analyses were done on the batch-corrected dataset. Data were log2 transformed after alignment and normalization. Data were uploaded and analyzed in the R2: Genomics Analysis and Visualization Platform, or analyzed in R.
Data analysis	Custom code for Ridge regression analyses was performed in R 4.0.3. Statistical analysis was done using GraphPad Prism version 9.3.1, Genomics Analysis and Visualization Platform R2 or R. Statistical tests are indicated in legends, were performed two-sided with p < 0.05 considered significant and indicated with; *p < 0.05, **p <0.01, ***p < 0.001, **** p < 0.00001. Error bars show the SD of the mean. Spearman correlation was determined with p < 0.05 considered significant. The Kaplan–Meier method was used to assess OS along with the log-rank test for statistical significance in R2 (patient data) or GraphPad (mice data).

For manuscripts utilizing custom algorithms or software that are central to the research but not yet described in published literature, software must be made available to editors and reviewers. We strongly encourage code deposition in a community repository (e.g. GitHub). See the Nature Portfolio guidelines for submitting code & software for further information.

Data

Policy information about availability of data

All manuscripts must include a data availability statement. This statement should provide the following information, where applicable:

- Accession codes, unique identifiers, or web links for publicly available datasets
- A description of any restrictions on data availability
- For clinical datasets or third party data, please ensure that the statement adheres to our policy

Materials availability: All unique/stable reagents generated in this study are available from the lead contact with a completed materials transfer agreement.

Data and code availability
• RNA-Seq data have been deposited at Gene Expression Omnibus (GEO) and is publicly available as of the date of publication.
• All the R scripts used in this study are available upon request and without restriction to the lead contact (m.f.bijlsma@amsterdamumc.nl)
• Any additional information required to reanalyze the data reported in this work paper is available from the lead contact upon request.

Human research participants

Policy information about studies involving human research participants and Sex and Gender in Research.

Reporting on sex and gender	For all esophageal patient samples, biological sex is reported. Sex was determined based on the medical record of patients. The sex distribution of esophageal cancer incidence is 80% in man versus 20% in woman, which was similar in our cohort.
Population characteristics	Patient characteristics were similar to population characteristics of EAC patients. Details of the characteristics of the cohort to be found in Supplemental table 1. Patient characteristics of EAC pre-treated biopsies.
Recruitment	Eligible patients from the outpatient clinical of the Amsterdam UMC were ≥ 18 years with pathologically confirmed EAC. Gastroesophageal junction tumors were eligible if the bulk of the tumor was located in the distal esophagus or on the gastroesophageal junction. All patients provided written, informed voluntary consent for study participation. This study was conducted in accordance with the Declaration of Helsinki and the international standards of good clinical practice.
Ethics oversight	Study portocol was approved by the Medical ethical committee (BiOES; METC 2013_241).

Note that full information on the approval of the study protocol must also be provided in the manuscript.

Field-specific reporting

Please select the one below that is the best fit for your research. If you are not sure, read the appropriate sections before making your selection.

☒ Life sciences ☐ Behavioural & social sciences ☐ Ecological, evolutionary & environmental sciences

For a reference copy of the document with all sections, see nature.com/documents/nr-reporting-summary-flat.pdf

Life sciences study design

All studies must disclose on these points even when the disclosure is negative.

Sample size	The sample size calculation for the cell lines comparing a slow versus fast onset of EMT (2-sample, 2-sided equivalence) is as follows: Required significance level is 0.05, 90% power; n=11 total samples. Adding a 10% dropout rate, this resulted in 12 cell line samples.<br>For the number of samples required for RNA-Seq analysis, formal group size calculations were difficult, so this was based on previous RNA-Seq analyses on pancreatic ductal adenocarcinoma, where in 90 specimens we have been able to identify molecular subtypes with strong prognostic power.
Data exclusions	Exclusion criteria for RNA-seq of patient samples were: <18 years of age and another active malignancy interfering with the prognosis of esophageal adenocarcinoma. After RNA-Seq, GAC and ESC samples were excluded to obtain a group of solely 78 EAC samples for analyses.
Replication	Replicates are indicated in the legends.
Randomization	Mice were randomized over the different treatment groups.
Blinding	The timing of appearance of mesenchymal morphology based on brightfield images was assessed by three independent and blinded assessors (Cohen's kappa p<0.001; Fig. 1c).

Reporting for specific materials, systems and methods

We require information from authors about some types of materials, experimental systems and methods used in many studies. Here, indicate whether each material, system or method listed is relevant to your study. If you are not sure if a list item applies to your research, read the appropriate section before selecting a response.

Materials & experimental systems

n/a	Involved in the study
☐	☒ Antibodies
☐	☒ Eukaryotic cell lines
☒	☐ Palaeontology and archaeology
☐	☒ Animals and other organisms
☐	☒ Clinical data
☒	☐ Dual use research of concern

Methods

n/a	Involved in the study
☒	☐ ChIP-seq
☐	☒ Flow cytometry
☒	☐ MRI-based neuroimaging

Antibodies

Antibodies used	E-Cadherin, 1:200, Cat. No: 324105, BioLegend, EpCAM, 1:200, Cat. No: 324243, BioLegend, CXCR4,1:200, Cat. No: 306515, BioLegend, N-Cadherin, 1:200, Cat. No: 350811, BioLegend, anti-human CD29, 1:200, Cat. No: 303014, BioLegend, mouse anti-vimentin (Santa Cruz, sc-73259, 1:300, anti-E-cadherin Abcam, Ab40772, 1:300, rabbit anti-laminin (Thermo Fisher, PA5-22901, 1:200, Alexa Fluor 448 anti-rabbit IgG1 (H+L, Invitrogen, A11008, 1:400) and Alexa Fluor 546 anti-mouse IgG (H+L, Invitrogen, A11030, 1:400).
Validation	Each antibody was titrated to obtain the optimal dilution for staining before experiments were performed.

Eukaryotic cell lines

Policy information about cell lines and Sex and Gender in Research

Cell line source(s)	Primary EAC cell lines were previously established from resected patient material as described before 11. Primary cell lines were obtained and established in agreement with pertinent legislation, Declaration of Helsinki, and patient's informed consent. Publicly available EAC cell lines Flo1 (RRID: CVCL_2045), OE19 (RRID:CVCL_1622) and OE33 (RRID:CVCL_1622; ATCC, Manassas, VA)
Authentication	All cell lines were checked for autenticity by STR profiling
Mycoplasma contamination	All cell lines were checked for mycoplasma each month and results were negative.
Commonly misidentified lines (See ICLAC register)	*Name any commonly misidentified cell lines used in the study and provide a rationale for their use.*

Animals and other research organisms

Policy information about studies involving animals; ARRIVE guidelines recommended for reporting animal research, and Sex and Gender in Research

Laboratory animals	NOD.Cg-Prkdcscid Il2rgtm1Wjl / Szj (NSG) mice, 12 weeks of age
Wild animals	No wild animals were used in this study.
Reporting on sex	Both males and females were used, equally distriuted over the treatment groups.
Field-collected samples	No field-collected samples were used in this study.
Ethics oversight	Mice animal work procedures were approved by the animal experimental committee of the institute according to Dutch law and performed in accordance with ethical and procedural guidelines established by the Amsterdam UMC, location AMC and Dutch legislation. Ethical approval number was AVD1180020171672.

Note that full information on the approval of the study protocol must also be provided in the manuscript.

Clinical data

Policy information about clinical studies

All manuscripts should comply with the ICMJE guidelines for publication of clinical research and a completed CONSORT checklist must be included with all submissions.

Clinical trial registration	No clinical trial was performed for this study.
Study protocol	All patient material was collected with consent under ethical approval (METC 2013_241).
Data collection	Snap frozen esophageal tumor biopsy samples were collected in the Amsterdam UMC between May 22 2013 till June 1 2020 with ethical approval (BiOES; METC 2013_241).
Outcomes	Although this was not a clinical study, our primary outcome measure was overall survival, secondary: occurence of metastases

Flow Cytometry

Plots

Confirm that:

☒ The axis labels state the marker and fluorochrome used (e.g. CD4-FITC).

☒ The axis scales are clearly visible. Include numbers along axes only for bottom left plot of group (a 'group' is an analysis of identical markers).

☒ All plots are contour plots with outliers or pseudocolor plots.

☒ A numerical value for number of cells or percentage (with statistics) is provided.

Methodology

Sample preparation	Cells were harvested using trypsin-EDTA (Lonza) and washed in FACS buffer (1% FCS in PBS). Cells were stained for 30 minutes at 4°C with the following antibodies diluted in FACS buffer; anti-human CD324 (E-Cadherin, 1:200, Cat. No: 324105, BioLegend), anti-human CD326 (EpCAM, 1:200, Cat. No: 324243, BioLegend), anti-human CD184 (CXCR4,1:200, Cat. No: 306515, BioLegend), anti-human CD325 (N-Cadherin, 1:200, Cat. No: 350811, BioLegend), and anti-human CD29 (1:200, Cat. No: 303014, BioLegend). Intracellular epitopes were targeted using permeabilization buffer (BD Biosciences, San Jose, CA).
Instrument	BD FACSCanto
Software	Data were analyzed using FlowJo 10 (Tree Star, Ashland, OR).
Cell population abundance	Cell population abundance was depended per experiment and cell line.
Gating strategy	Gating strategy for primary cell lines to obtain a clean tumor population was performed with a FACS-sort of FITC (488 nm) with FITC 530/30 vs APC 660/20, see STAR protocol PMID: 36825807. Gating strategy for mesenchymal or epithelial markers was geometric mean fluorescence (gMFI) intensity in the relevant channel, corrected for isotype control, yielding the ΔgMFI.

☐ Tick this box to confirm that a figure exemplifying the gating strategy is provided in the Supplementary Information.
